# Value of C-reactive Protein/Albumin Ratio for Predicting Ischemia in Myocardial Perfusion Scintigraphy

**DOI:** 10.4274/mirt.galenos.2020.88261

**Published:** 2020-10-19

**Authors:** Süleyman Çağan Efe, Özlem Özdemir Candan, Cihan Gündoğan, Ahmet Öz, Yasin Yüksel, Burak Ayca, Tevfik Fikret Çermik

**Affiliations:** 1 Kartal Koşuyolu Cardiovascular Diseases Training and Research Hospital, Clinic of Cardiology, İstanbul, Turkey; 2 İstanbul Training and Research Hospital, Clinic of Internal Medicine, İstanbul, Turkey; 3 İstanbul Training and Research Hospital, Clinic of Nuclear Medicine, İstanbul, Turkey; 4 İstanbul Training and Research Hospital, Clinic of Cardiology, İstanbul, Turkey

**Keywords:** Myocardial perfusion scintigraphy, stable coronary artery disease, C-reactive protein/albumin ratio

## Abstract

**Objectives:**

Several studies demonstrate the relationship between coronary artery disease and inflammatory parameters. Nevertheless, there is paucity of data regarding the role of high sensitivity (hs)-C-reactive protein (CRP) to albumin ratio (CAR) in patients with ischemia on gated single photon emission tomography (SPECT) myocardial perfusion imaging (MPI). This study was aimed at demonstrating the relationship between CAR and the occurrence of ischemia on gated SPECT MPI.

**Methods:**

We retrospectively evaluated 2.048 referred patients for gated SPECT MPI from a cardiology outpatient clinic between October 2017 and June 2019. After applying exclusion criteria and measuring serum CRP and albumin levels, we included 126 patients in the study. We then classified subjects into different groups according to the absence or presence of ischemia on gated SPECT MPI.

**Results:**

According to laboratory findings, hs-CRP and CAR were significantly higher in the ischemia group, while the serum albumin was significantly lower in ischemia group (p<0.05 for each). The independent predictors of presence of ischemia in multivariate analysis were hypertension and CAR (CAR; odds ratio: 5.720, 95% confidence interval: 2.697-12.133, p<0.001). The optimal value of CAR for presence of ischemia was 0.96 with 76% sensitivity and 71% specificity.

**Conclusion:**

We found CAR values as a predictor for ischemia before MPI.

## Abstract (tr)

**Amaç:**

Birçok çalışmada, koroner arter hastalığı ile enflamatuvar parametreler arasında ilişki bulunduğu gösterilmiştir. Bununla birlikte, tek foton emisyon tomografisi (SPECT) miyokard perfüzyon görüntülemesinde (MPI) iskemi bulunan hastalardaki C-reaktif proteinin (CRP)/albümin oranının (CAR) rolü ile ilgili yeterli veri bulunmamaktadır. Bu çalışmada, SPECT MPI’da CAR ve iskemi varlığı arasındaki ilişkiyi araştırmayı amaçladık.

**Yöntem:**

Ekim 2017 ve Haziran 2019 tarihleri arasında kardiyoloji polikliniğinden yönlendirilerek SPECT MPI yapılan 2,048 hasta retrospektif olarak değerlendirildi. Tarama popülasyonu değerlendirildiğinde serum CRP ve albümin düzeyleri ölçülen ve dışlama ölçütlerini karşılamayan 126 hasta çalışma grubunu oluşturdu. SPECT MPI’da iskemi varlığı ve yokluğuna göre hastalar iki gruba ayrılarak veriler değerlendirildi.

**Bulgular:**

Laboratuvar bulguları açısından değerlendirildiğinde iskemi bulunan grupta CRP ve CAR anlamlı olarak daha yüksek iken, bu grupta serum albümini anlamlı olarak daha düşük bulundu (her biri için p<0,05). Çok değişkenli analizde, iskemi varlığının bağımsız prediktörleri hipertansiyon ve CAR idi (CAR; göreceli olasılıklar oranı: 5,720,%95 CI: 2,697-12,133, p<0,001). İskemi varlığı için en uygun CAR değeri %76 hassasiyet ve %71 özgüllük ile 0,96 olarak bulunmuştur.

**Sonuç:**

Çalışmamız miyokard iskemisi ve CAR değerleri arasındaki ilişkiyi araştıran ilk çalışma olarak değerlidir. Miyokard perfüzyon sintigrafisi öncesi bakılan CAR değerleri iskemi için prediktör olarak bulundu.

## Introduction

In daily practice, many patients with chest pain and similar cardiac complaints are evaluated in outpatient clinics. Appropriate patients are oriented to perform stress electrocardiography, stress echocardiography, or gated single photon emission computed tomography (SPECT) myocardial perfusion imaging (MPI) to investigate myocardial ischemia. In addition, MPI is known to evaluate the prevalence and severity of myocardial ischemia (1,2).

Inflammatory process has an important role in atherosclerosis, plaque development, and plaque progression. High-sensitivity C-reactive protein (hs-CRP), neutrophile count, lymphocyte count, monocyte count, and albumin are the most studied and clinically evaluated inflammatory markers (3,4). Inflammatory markers can be used to determine patients’ risk and improving their clinical outcomes. Published studies on the prevention of cardiovascular diseases (CVD) highlight the availability of hs-CRP levels for determining risk stratification in selected patients (5). Adverse cardiovascular outcomes and prevalence of atherosclerosis have a significant association with elevated CRP levels (6). There is also an association with coronary artery disease (CAD) and other acute phase reactants like albumin. The fact that CRP and albumin are easily accessible markers in daily practice increases the frequency of using these markers. The CRP to albumin ratio (CAR) is a new marker, which is calculated by the ratio of these acute phase reactants. CAR is thought to be better at predicting inflammatory events (7,8,9). This study was aimed to investigate the predictability of ischemia from inflammatory markers, before performing MPI in stable CAD patients.

## Materials and Methods

**Figure 1:**
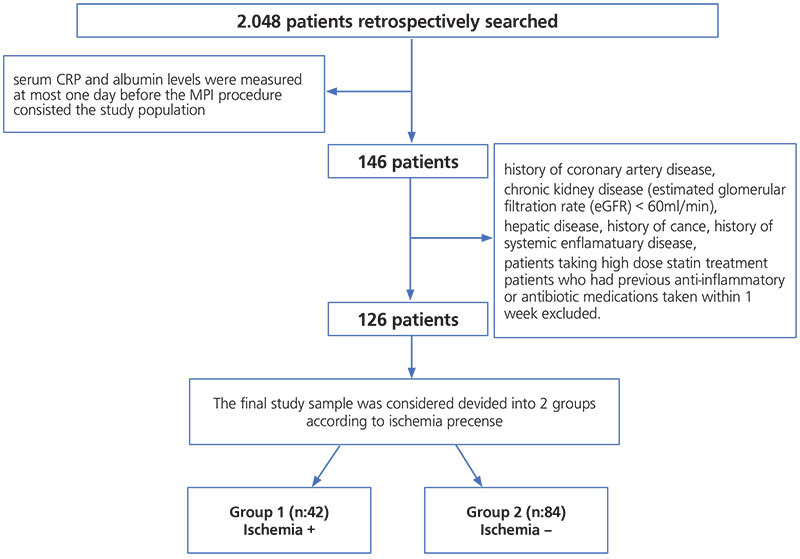
Study population flow chart CRP: C-reactive protein, MPI: Myocardial perfusion imaging, eGFR: Glomerular filtration rate

We retrospectively evaluated 2048 patients referred for gated SPECT MPI from a cardiology outpatient clinic between October 2017 and June 2019. Out of these, we enrolled patients with serum CRP and albumin levels measured one day before MPI. After applying exclusion criteria, we included 146 patients in the study. We contacted patients through telephone to obtain cardiovascular risk factors and diseases that might affect acute phase reactants. We further excluded participants having a history of CAD (with revascularization), chronic kidney disease (estimated glomerular filtration rate <60 mL/dk), hepatic disease (aspartate aminotransferase, alanine aminotransferase two-fold above normal limits), history of cancer or systemic inflammatory disease, patients taking statins, anti-inflammatory drugs, or antibiotic medications within one week from the study. Finally, we had 126 eligible patients for the analysis (Figure 1). The nuclear medicine department and myocardial perfusion studies (MPS) reports confirmed that all patients had taken dipyridamole or adenosine as a pharmacological stressor. We obtained a written patient informed consent. The Local Ethics Committee approval from İstanbul Training and Research Hospital with decision number 1.935 was taken for the present study.

### Myocardial Perfusion Imaging

We performed a two-day stress/rest imaging protocol using Technetium 99-m methoxy-isobutylisonitrile (Tc-99m MIBI) in order to evaluate myocardial perfusion. During peak exercise, we injected radiopharmaceutical agents on modified Bruce protocol or at peak hyperemia. For stress imaging, we used dipyridamole (0.142 mg/kg/min) or adenosine (0.28 mg/min) infusion as a radiopharmaceutical agent. Imaging began 30-45 min after injecting 15-20 mCi Tc-99m MIBI. We injected a similar dose for rest imaging if there was any suspected perfusion defect on stress images.

### SPECT Imaging Protocol

We obtained all images over a 180° angle orbit from right anterior oblique, 45° angle to left posterior oblique, 45° angle using a dual-head γ-camera (General Electric Optima NM/CT 640, GE Healthcare, Wauwatosa, WI, USA) equipped with ultra-high resolution collimator, 64x64 matrix, an elliptic orbit with step and shoot acquisition at 3° intervals over 180° angle, 60 projections and 9-13 s per projection using a 20% energy window centered on the 140 keV photopeak of Tc-99m. The patients lay in supine position on the surface with their arms raised straight above the head. Image sets obtained by SPECT analysis were reconstructed on a dedicated workstation (Xeleris, GE Healthcare, Haifa, Israel) using WBR and Evolution for Cardiac recommended manufacturer relative risk and noise reduction parameters, with and without CT based AC (12 iterations and 10 subsets). At the end of each acquisition, a single low-dose CT scan (100 keV; 1.0 mA; 0.2-0.3 mS) of the chest was performed to obtain attenuation maps automatically applied by the processing software in order to correct the emission data. The MPI dataset was carefully re-matched with the CT attenuation map to produce the attenuation corrected images.

### Laboratory Measurements

We performed routine blood chemistry measurements including hs-CRP (mg/L) in the morning. In addition, we measured albumin and hs-CRP levels using Roche Diagnostics Cobas 8000 analyzer. We deduced the CAR (mg/g) value by dividing hs-CRP level to albumin level, and results were obtained from the same blood samples.

### Statistical Analysis

We performed overall analysis using Statistical Package for Social Sciences (SPSS statistical version 17.0 Inc, Chicago, IL). We described continuous variables as mean ± standard deviation or median values, while categorical variables as frequencies and percentages (%). We verified the normality of continuous variables using Kolmogorov-Smirnov statistics. Meanwhile, we performed Mann-Whitney U test and independent t-test to evaluate parametric variables. We used χ2-test or Fisher’s Exact test to evaluate categorical variables. Statistical significance was set at p<0.05. The variables that showed marginal association in univariate analysis were moved to multivariate logistic regression analyzes. We performed multivariate logistic regression analyzes to obtain independent predictors of ischemia in MPI. The receiver operating characteristics curve analysis was used for calculating the CAR value that predicts presence of ischemia with the best possible specificity and sensitivity.

## Results

**Figure 2:**
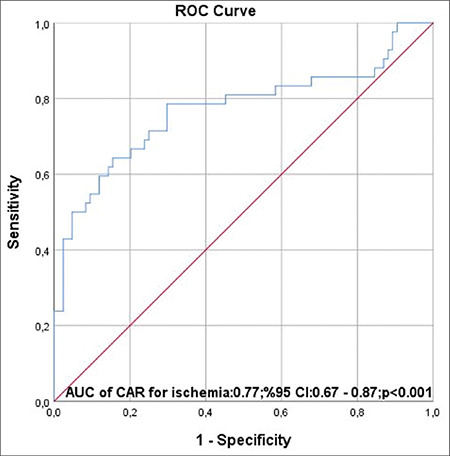
ROC curve analysis ROC: Receiver operating characteristics, AUC: Area under curve, CAR: CAR: C-reactive protein/albumin ratio, CI: Confidence interval

All participants in the study underwent gated SPECT MPI. The mean age of participants was 59.8±11.3 years and 56 patients (44%) were male. The subjects were classified into groups according to the absence or presence of ischemia on gated SPECT MPI. Table 1 shows the demographic characteristics of all the study population. The frequencies of diabetes mellitus and history of hypertension were significantly more frequent in patients with ischemia (p<0.05 for each). Based on laboratory findings (Table 2), hs-CRP and CAR were significantly higher in the ischemia group, but serum albumin was significantly lower in this group (p<0.05 for each). Other laboratory findings were not different between the groups. Hypertension, diabetes mellitus, and CAR were the predictors of presence of ischemia in univariate analysis (Table 3). From multivariate regression analysis using a model adjusted for the aforementioned parameters, hypertension [odds ratio (OR): 4.012, 95% confidence interval (CI): 1.580-10.118, and p=0.003] and CAR (OR: 5.720, 95% CI: 2.697-12.133, and p<0.001) were independently correlated with the presence of ischemia. The area under curve of CAR for gated SPECT MPI in the ischemia group was 0.77 (0.67-0.87 95% CI, p<0.001) (Figure 2). Moreover, the optimal value of CAR in the group with the presence of ischemia was 0.96 (sensitivity=76%; specificity=71%).

## Discussion

In this study, we evaluated the association between CAR and MPI detected ischemia in patients with stable CAD. Among various inflammatory parameters, CAR showed the best capability to discriminate myocardial ischemia detected by MPI. This association was independent of other predictors.

Hs-CRP is considered to be an independent cardiovascular event marker used in cardiovascular risk classification, and in some scores like Reynolds score, hs-CRP is present (3,10). Redberg et al. (6) hypothesized that hs-CRP is both an inflammatory marker and a predictor of an increased risk of CVD. As a result, there is a clear relationship between increased hs-CRP levels and higher CV risk (11,12).

There is an ongoing debate on whether hs-CRP is an indicator of inflammation or whether it contributes to the development of atherosclerosis (6). In atherosclerotic plaques, elevated hs-CRP levels were associated with increased number of monocytes, increased vascular endothelial plasminogen actıvator inhibitor-1 expression, and reduced low density lipoprotein uptake by tissue macrophages. The hs-CRP is a known cause of oxidative stress, resulting in remodeling of the vascular wall through angiotensin activity increase. It also activates the prothrombotic states in blood. There is evidence that demonstrates the presence of abundant CRP in atherosclerotic lesions and also defines CRP as the trigger for various pathogenic pathways causing atherogenic events (13). Plasma level of hs-CRP is known to increase with the complexity of CAD (5). Hs-CRP can be used as a predictor of CVD, ischemic stroke, and myocardial infarction (14). Increased CRP at admission in patients with unstable angina or non-ST-segment myocardial infarction is associated with increased 14-day mortality, even in patients with a negative rapid cardiac troponin test (15). In other studies with stable CAD patients treated with PCI, CRP independently associated with an increased risk of 1-year mortality (16). In STEMI patients, CAR was found to be effective as prognostic marker for in-hospital and long-term all-cause mortality (17). As already known, our study determined the relationship between CRP and myocardial ischemia.

Albumin is known as an acute phase marker with reduced levels in cases of infection and chronic inflammation. Low albumin levels are known to have prognostic significance in cases such as acute coronary syndrome, heart failure, and stable coronary heart disease (18). Hypoalbuminemia is also known as an independent prognostic indicator in many CVDs (19). In our study, low albumin levels were associated with ischemia.

It is well-known that inflammatory reactions take place in all stages of myocardial ischemia. In ischemic myocardial tissue, pro-inflammatory chemokines are strongly secreted thereby activating leukocytes leading to leukocyte migration to the inflammation area. A growing set of clinical evidence suggests that neutrophil-to-lymphocyte ratio (NLR), CRP, albumin, and lymphocyte are easily accessible and cheap markers to assess inflammation, especially in acute coronary syndromes (20). NLR was found as an independent predictor for acute and stable CAD in some studies (21,22). In a study where CAR values were compared with other inflammatory parameters, CAR was found to be a better predictor than other parameters for significant CAD in patients with stable CAD (23). Despite previous studies, NLR was not associated with myocardial ischemia in our study.

Several reports show that the severity and extent of myocardial ischemia can be determined accurately using SPECT MPI; they have also highlighted the prognostic value of this method. Spectral perfusion screening has been reported to have lower CRP levels in patients with normal myocardial functions than with myocardial ischemia (24).

The CAR is a marker that is calculated by the ratio of these acute phase markers; and is believed to be more sensitive in predicting inflammatory events. In some CVD (such as stable angına pectoris and acute coronary syndromes), CAR values were higher than in control groups and disease severity was associated with CAR values (7,8,9). In our study, ischemia group in MPS had significantly higher CAR values than those without ischemia.

Although CRP and albumin are both acute phase markers, there is experimental evidence that demonstrates the presence of CRP in atherosclerotic lesions. The exertion and measurable values of these markers vary slightly in patients with ischemia due to myocardial ischemia in daily life efforts. Our study is a novel one which investigates the relationship between myocardial ischemia and CAR values. Therefore, CAR values at any time in patients with stable CAD may be predictive for ischemia detection in outpatient clinics.

### Study Limitations

Owing to the retrospective nature of this study, we could not assess myocardial viability by MPI, and hence could determine the ischemia percentage from MPI reports.

## Conclusion

CAR is an independent variable in detecting myocardial ischemia in stable CAD patients. CAR values can be used to predict the presence of ischemia prior to invasive procedures in daily in daily practice.

## Figures and Tables

**Table 1:**
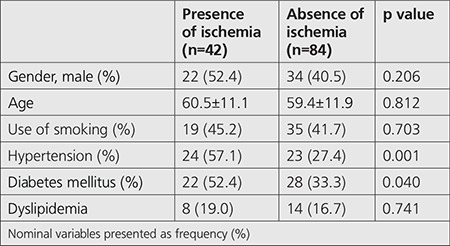
Baseline demographic characteristics findings of all patients

**Table 2:**
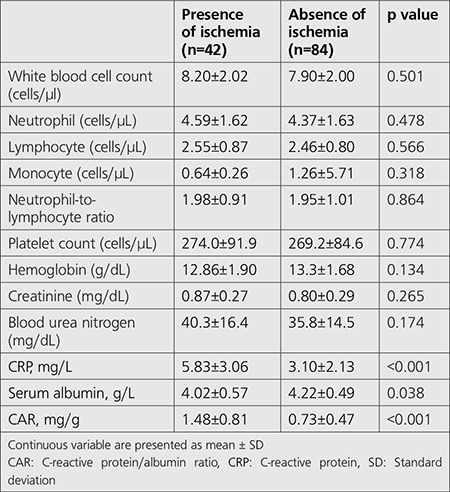
Laboratory findings of all patients

**Table 3:**
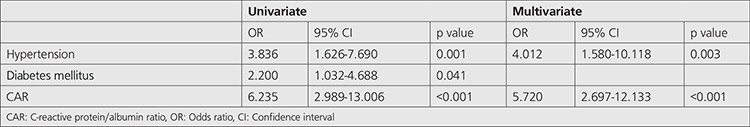
Uni-multivariable logistic regression analysis for presence of ischemia
